# Outcome of biventricular repair of congenital cardiac malformations with a borderline left ventricle

**DOI:** 10.1186/s13019-026-04531-w

**Published:** 2026-07-17

**Authors:** Emilie Houdebert, Victoria Benito Arnaiz, Antonio Gonzalez-Calle, Mustafa Rady, Giulia Scopetani, Lorenzo Boni, Maria-Helena Perez, Nicole Sekarski, Stefano Di Bernardo, Andrew Parry, Amir-Reza Hosseinpour

**Affiliations:** 1 Faculty of Biology and Medicine, University of Lausanne, Lausanne, Switzerland; 2 Congenital Cardiac Surgery Unit, 12 de Octubre University Hospital of Madrid, Madrid, Spain; 3 Congenital Cardiac Surgery Unit, Virgen del Rocio Hospital, University Hospitals of Seville, Seville, Spain; 4 Department of Cardiac Surgery, Bristol Children’s Hospital, Bristol, UK; 5 Service of Cardiac surgery, Lausanne University Hospital and University of Lausanne, Lausanne, Switzerland; 6 Pediatric Intensive and Intermediate Care Units, Service of Pediatrics, Department of Women-Mother-Child,, Lausanne University Hospital and University of Lausanne, Lausanne, Switzerland; 7 Pediatric cardiology unit, Service of Pediatrics, Department of Women- Mother-Child, Lausanne University Hospital and University of Lausanne, Lausanne, Switzerland; 8 Faculté de Biologie et de Médecine de Lausanne, Université de Lausanne, Lausanne, CH-1015 Switzerland

**Keywords:** Borderline left ventricle, Borderline LV, Biventricular repair, Outcome

## Abstract

**Introduction:**

Biventricular repairs are occasionally done on hearts with a borderline-sized left ventricle to avoid univentricular palliation. We aim to assess the outcome.

**Materials and methods:**

Data of 27 patients with borderline left ventricle that had undergone biventricular repair in our centers in the past 20 years (median follow-up: 5 years) were analyzed retrospectively.

**Results:**

Successful primary repair was performed in 17 (63%) patients. Four (15%) patients initially underwent primary repairs, which were converted to Norwood reconstructions, hoping to re-attempt biventricular repair later. Primary Norwood reconstruction with a restrictive atrial septal defect or its hybrid equivalent were performed in 6 patients (2 Norwoods [7%], 4 Hybrids [15%]) for ventricular rehabilitation, with subsequent biventricular repair a few months later. The total number of procedures was 87 (average: 3.2 per patient). These figures do not include procedures for implantation of ECMO, re-operations for bleeding and delayed sternal closures because we consider these as parts of the procedures that immediately preceded them. This strategy failed in 8 (30%) patients: 7 died (4 within 1 year, 1 at 18 months, 1 at 3 years, 1 at 3.5 years), and 1 was converted to univentricular palliation eventually reaching Fontan completion. Four (15%) patients reached favorable outcomes: alive and asymptomatic with biventricular circulation, good ventricular function and normal-sized valves. The remaining 15 (55%) patients are alive but with residual lesions, which may include ventricular diastolic dysfunction, somewhat small mitral and/or aortic valves and pulmonary hypertension; 9 are in NYHA class I and 6 in class II.

**Conclusion:**

The results are suboptimal. Further studies are needed to identify ways of predicting who would benefit most from aggressively pursuing a biventricular strategy and offer it to them selectively.

**Supplementary Information:**

The online version contains supplementary material available at https://doi.org/10.1186/s13019-026-04531-w.

## Introduction

In the last 2 decades, a tendency has emerged to favor biventricular repair strategies over univentricular palliation for congenital cardiac malformations in which the left ventricle (LV) is only slightly too small to qualify as normal––so-called *borderline LV*––although the criteria for such a diagnosis have always lacked precision and varied both between centers and even within the same centers over the years [1–8]. Such a strategy may be a multi-stage approach starting with procedures that may stimulate LV growth before the actual biventricular repair, or a single-stage repair if the LV is considered of sufficient capacity for this and that it may grow more by functioning within a biventricular circulation. Essentially, this is a strategy of pushing the limits of biventricular repair to avoid univentricular palliation, thus avoiding a Fontan circulation. This approach has gained favor in response to the well-known morbidity of the Fontan circulation (multi-organ dysfunction) as well as its long-term mortality (15% at 25 years in one study and 25% at 20 years in two studies, although this increases to 60% at 30 years) [9–13]. Biventricular repair is offered with the expectation that the outcome would be better even if the LV is slightly small initially. Such enthusiasm for biventricular repair may be increased by recent data suggesting that Fontan outcomes are even worse when the rudimentary ventricle is larger than 50% of normal size (which applies to all borderline LVs), likely because a relatively large rudimentary ventricle may interfere with and undermine the function of the dominant ventricle [14, 15].

However, congenital cardiac malformations with a borderline LV may not lend themselves easily and safely to biventricular repair for many reasons. First, the operations required to achieve a biventricular repair may be complex and high-risk in certain cases. Second, such ventricles may not necessarily grow as we may expect or hope. Third, even if such ventricles do grow to a normal size, their function may not necessarily develop equally well such that they may not respond well to the normal volume and pressure loadings of a biventricular circulation [2–8]. In other words, such ventricles may suffer from diastolic dysfunction, which may eventually also lead to systolic dysfunction in the long term, thus compromising outcome. Fourth, a borderline LV usually has borderline-sized mitral and aortic valves, which must also grow, otherwise the outcome will not be good. However, valves may not necessarily grow in keeping with their ventricle; we know this from studies of the tricuspid valve and right ventricular size in patients with pulmonary atresia and intact ventricular septum showing that tricuspid valve size is the main limiting factor in achieving a biventricular repair [16–19]. Aortic or mitral valves of inadequate size can compromise outcome just as easily as LV size/function can. Finally, if biventricular repair is done but not tolerated well, the only option is to convert to univentricular palliation, but the outcome may not be as good as when univentricular palliation is offered primarily, since the imposed biventricular circulation may have damaged the pulmonary vasculature.

These doubts and concerns prompted us to retrospectively analyze and report the outcomes of our patients with a borderline LV that were treated with a biventricular repair strategy.

## Patients and methods

This is a retrospective observational study approved by our ethics’ committee (Reference: 2025 − 00992, granted on May 26, 2025), aiming to evaluate the outcome of biventricular repair in patients with a borderline LV.

The methodology of this study must start by explaining what we mean by a borderline LV. First and foremost, this term was not defined prospectively by any of the centers involved in this work, since the decisions were made on an individual basis as part of their daily clinical work with no plan to construct any study, especially given that each center has done very few such cases spread over a long period (20 years). Rather, this term is applied retrospectively for this study to unify all patients from 4 centers. In fact, the criteria used for this diagnosis varied in each center, and even within the same center but for different patients, and these were not always rigorously recorded. Furthermore, in some cases, the diagnosis of borderline LV was made subjectively based on the size of the LV itself and/or that of the mitral or aortic valves without providing any numerical data. Nevertheless, we accepted those subjective evaluations for two reasons. First, these decisions were made after considerable deliberation in several multidisciplinary meetings for each patient involving all principal members of the care team reviewing all imaging data (mainly echocardiographic and catheterization data in earlier cases, but also cardiac magnetic resonance imaging more recently). Second, we trust that numerical data were likely discussed at the time in those multidisciplinary meetings even though these were not recorded. When numerical data were recorded, these included 1–3 of the following 4 features:

- LV diastolic volume < 20 ml/m^2^.
- LV diastolic diameter with a Z-score < -2.5.
- Non-apex-forming LV, with a left/right ventricular length ratio around 0.8.
- Mitral and/or aortic valve diameters with Z-scores around − 2.5.

Therefore, for the purposes of this study, the diagnosis of borderline LV reflects the diagnoses made in each individual center, trusting that the evaluation was done with due rigor even if the only information available may be subjective in some cases. Nevertheless, the criteria used in each case are outlined in the Supplementary File––Table 1.

**Table 1:** Outcomes of biventricular repairs in patients with a borderline left ventricle

	Number of patients	Total number of procedures	Outcomes
**Primary repair**	17 (63%)	35	- 3 died at 6, 9 and 18 months due to severe LV dysfunction in 2, and sudden death in the presence of LV outflow obstruction and mitral stenosis in 1
			- 2 are alive with favorable outcomes at 9 & 13 years’ follow up
			- 12 are alive but with at least one of the following residual lesions: LV dysfunction in 7, mitral stenosis in 10, LV outflow stenosis in 8
**Primary repair converted to Norwood**	4 (15%)	19	- 2 died in the neonatal period after the Norwood procedure (multi-organ failure)
			- 1 converted to Fontan pathway, now living with Fontan circulation but has hepatopathy at 20 years follow-up
			- 1 converted back to biventricular repair: alive with favorable outcome at 7 years’ follow-up
**Primary Norwood**,** Later repair**	2 (7%)	8	- 1 died at age 3 years after staged biventricular conversion and re-conversion back to univentricular, which failed due to LV dysfunction
			- 1 is alive with favorable outcome at 6 years’ follow-up
**Primary hybrid**,** later repair**	4 (15%)	25	- 1 died at age 3 years following surgery for the final stage of biventricular repair (LV dysfunction)
			- 3 are alive but with residual lesions: LV dysfunction in 2, mitral stenosis in 3, LV outflow stenosis in 1,
**Total**	27	87	- 8 (30%) failures: 7 died, 1 is alive with a Fontan circulation and hepatopathy
			- 4 (15%) are alive with favorable outcomes
			- 15 (55%) are alive but with ventricular dysfunction and/or small valves

### Inclusion criteria

All patients with a diagnosis of borderline LV who had undergone biventricular repair in our centers in the past 20 years, i.e. from 2005 to 2025. These patients are presented in Supplementary File––Table 1, which includes the criteria used for the diagnosis of a borderline LV in each case. The size assessments must have been based on images obtained after the 3rd day of life to ensure the ventricle was adequately loaded.

### Exclusion criteria

If assessments of the above criteria were based on images obtained in the first 3 days of life, which may result in falsely low values due to ventricular underloading.

A total of 27 patients (13 boys, 14 girls) met our criteria and were included in this study. Of the whole cohort, 26 patients had coarctation of the aorta with aortic arch hypoplasia and a borderline LV, and 1 had a borderline LV but with a normal aorta (detailed in supplementary file––Table 1). In all patients, the first operation was done in the neonatal period. These patients were followed-up for a median of 5 years (range: 1 month – 20 years, interquartile range: 3–12 years). Patients’ data were obtained from their electronic medical records, specifically searching for the following primary and secondary endpoints:

Primary endpoints:

1. The number and nature of interventions: procedures in preparation for biventricular repair, the repair itself, and subsequent procedures for addressing residual or recurrent lesions. These figures do not include procedures for implantation of postoperative ECMO, re-operations for bleeding and delayed sternal closures because we consider these as parts of the procedures that immediately preceded them.
2. Failure of biventricular repair. This is defined as one of 3 events: death, conversion to univentricular palliation with a view to proceeding towards a Fontan circulation, or heart transplantation. Note that palliative procedures used as intermediary measures to prepare the heart for future biventricular repair do not count as failures provided that biventricular repair is eventually performed successfully; otherwise, these would also count as failures.
3. Favorable outcome. This is defined as being alive and asymptomatic with biventricular circulation, good ventricular function, and normal-sized valves. Here again, we depend on subjective assessments of each center as these parameters are not clearly indicated numerically in the medical records especially since many of them were studied only by echocardiography.

Secondary endpoints: Residual LV diastolic dysfunction, residual mitral stenosis, residual LV outflow tract obstruction, and NYHA class, all at their latest follow-up. Here again, the information available in the medical records are largely subjective (non-numerical) based on echocardiography rather than catheterization in most cases.

## Results

Primary biventricular repair was successfully performed in 17 (63%) patients. Another 4 (15%) patients also received primary repairs in the first instance but did not tolerate it; these patients were placed on extracorporeal membrane oxygenator support (ECMO), and their repair converted to a Norwood reconstruction a few days later, maintaining the option of re-attempting biventricular repair at a later date. Primary Norwood reconstruction with a restrictive atrial septal defect (ASD) or its hybrid equivalent was done in 6 (22%) patients (2 Norwoods [7%], 4 hybrids [15%]) as part of a ventricular rehabilitation strategy with subsequent biventricular repair a few months later. The total number of procedures was 87, yielding an average of 3.2 per patient. These were 29 total repair procedures, 6 Norwoods, 4 hybrids, 22 percutaneous balloon dilatation of stenotic vessels (aortic or pulmonary), 1 surgical augmentation of the ascending aorta, 7 aortic valvar procedures (including 4 Ross-Konno procedures), 3 mitral valvar procedures, 1 tricuspid valve repair, 4 ASD closures, 2 superior cavopulmonary anastomoses, 1 Fontan completion, 2 hemi-diaphragmatic plications, 2 implantations of permanent pacemakers, 1 embolization of a major collateral artery, and 2 laparotomies for intestinal complications. Note that these figures do not include procedures for implantation of ECMO, re-operations for bleeding and delayed sternal closures because we consider these as parts of the procedures that immediately preceded them.

This strategy failed in 8 (30%) patients: 7 died and 1 was converted to the univentricular path, eventually undergoing Fontan completion. No patient received a heart transplant. The 7 deaths occurred within the first year in 4 cases, at 18 months in 1 case, at 3 years in 1 case, and at 3.5 years in 1 case. All 4 patients who died in the first year of life had undergone primary biventricular repairs, 2 of which were converted to Norwood reconstructions and died within a few days following this operation while the other 2 patients died of ventricular dysfunction and pulmonary hypertension 6 and 9 months following their biventricular repair. The patient who died 18 months following surgery had undergone a primary biventricular repair. The patient who died at 3 years had undergone a hybrid palliation first, followed by biventricular repair, and died of left ventricular dysfunction and outflow stenosis. The patient who died at 3.5 years had undergone a Norwood operation with a restrictive atrial septal defect followed by a superior cavopulmonary anastomosis with aortic arch augmentation, followed by biventricular conversion. This, however, was not tolerated and had to be converted back to univentricular physiology, requiring ECMO, but the patient never recovered. The patient who underwent a Fontan operation was also initially offered a primary biventricular repair, but this was not tolerated and was converted to a Norwood reconstruction followed by superior cavopulmonary anastomosis and later Fontan completion. He is now 20 years old and already suffers from Fontan-associated liver disease (FALD). These patients, including their causes of death, are presented in Table 1.

Among the 19 survivors of biventricular repair (70% of the cohort), the LV did grow to a normal size but not necessarily with a normal function, and the mitral and aortic valves have not grown to a normal size in most cases. Here again, we depend on subjective assessments of each center as these parameters are not clearly indicated numerically in the medical records; we trust them for the same reasons as outlined above and have recorded them in the Supplementary File––Table 1 with as much precision as available in the records. Specifically, only 4 patients (15% of the entire cohort) reached a favorable outcome, meaning that they are alive and asymptomatic with biventricular circulations, good ventricular function and normal-sized valves. Of these 4 patients, 2 had undergone primary biventricular repair and are now 9 and 13 years old, although percutaneous dilatation of re-coarctation was needed in one of them. One patient underwent an initial biventricular repair that was not tolerated and was converted to a Norwood reconstruction with a restrictive ASD to rehabilitate the left ventricle; biventricular repair was performed again 11 months later with favorable outcome, and she is now 7 years old. One patient underwent a Norwood operation with a restrictive ASD to rehabilitate the left ventricle, and subsequent biventricular repair with favorable outcome at 6 years’ follow-up.

The remaining 15 patients (55% of the cohort) are alive but with residual lesions. These patients represent 79% of survivors of biventricular repairs. Nine of these patients are in NYHA class I, and 6 in class II. The residual lesions, as per their latest follow-up, consist of at least 2 of the following 3 features:

- Diastolic dysfunction.
- Mitral valve stenosis with mean gradient of about 5–6 mmHg.
- Stenosis of the LV outflow tract with mean gradient of 16–40 mmHg.

These outcomes are presented in the Supplementary File––Table 1 and summarized in Table 1.

## Discussion

The results of biventricular repair strategy for hearts with a borderline LV in our series are suboptimal compared with the usual expectations from biventricular repair. Although this strategy does avoid the well-known late complications of a Fontan circulation (multi-organ dysfunction), it often leads to other complications that are also important including ventricular dysfunction and inadequate valvar size, which are likely to compromise long-term outcome. In fact, the failure rate in this series (defined as death, transplantation or definitive conversion to univentricular palliation) is high: 30% at a median follow-up of 5 years. Only 15% of patients reached a favorable outcome: alive and asymptomatic with a biventricular circulation and no residual lesions. The remaining 55% of patients (79% of survivors of biventricular repair) have residual lesions that will likely further undermine prognosis at longer follow-up.

These sobering findings may be a warning against forcing the indications of biventricular repair. Such a warning is not new; it first appeared in an article published in 1996 by the Great Ormond Street group in London comparing the short- and intermediate-term outcomes of complex biventricular repairs with those of the Fontan operation in patients with the same diagnoses (complex malformations but with two functional ventricles) [20]. They found that short- and intermediate-term morbidity and mortality were higher in the biventricular repair group than in the Fontan group. Specifically, their actuarial estimate of survival at 7 years was 68% in the biventricular repair group and 94% in the Fontan group (*p* = 0.048). They explained these findings by stating that the high risks and potential suboptimal anatomic results of complex biventricular repairs may, in certain circumstances, outweigh the long-term shortcomings of the Fontan circulation. This idea was also insinuated in a study from Boston, published in 2000, assessing the long-term outcome of biventricular correction of transposition of the great arteries with ventricular septal defect and LV outflow tract obstruction (the Rastelli operation) [21]. They reported a high reintervention rate and a transplant-free survival of only 52% at 20 years. They mentioned in their discussion that better outcomes may be expected with a Fontan circulation. Similar results for this malformation were reported in 2010 in a European multicenter study [22].

This dilemma of biventricular versus univentricular approach was extensively elaborated with complex statistical analyses and modelling in a publication in 2007 by the Toronto group [23]. They studied 362 patients with borderline left-sided structures and compared 5-year survival between those with univentricular palliation and those with biventricular repair. They concluded that inappropriate biventricular repair (i.e. doing biventricular repairs when we should not) has disastrous results whereas inappropriate univentricular palliation (i.e. offering univentricular palliation when biventricular repair is possible) is not ideal for patients but they live, i.e. Fontan is second best but not disastrous. They also reminded us that the early results of the Fontan procedure are very good and that problems arise 15–30 years later.

A further study from Boston, published in 2023, examined the outcomes of biventricular repair following various LV recruitment strategies in patients with a borderline LV [5]. These LV recruitment strategies were part of a multi-stage approach that included valvuloplasties, resection of endocardial fibroelastosis, and restriction of the atrial septal defect to augment LV filling and growth before eventual completion of the anatomic (biventricular) correction. They had proactively identified suitable candidates early to minimize delays in implementing these strategies. Nevertheless, their transplant-free survival without conversion to univentricular path was only 46% at a median follow-up of only 2.8 years. Even amongst successful cases (i.e. transplant-free survivors that did not require conversion to the univentricular path), reintervention rate was high: 35% during the same follow-up period.

These outcomes, like those we report, are far from ideal and all lead to the same question that whether biventricular repair strategies in such complex cases achieve their intended objective of resulting in better outcomes than with a Fontan circulation. This question is not easy to answer. Although these outcomes are far worse than the reported outcomes after Fontan operations, this comparison is not valid. The published Fontan series only report the outcomes of patients that had already reached a Fontan circulation; patients that may have died in earlier stages of palliation or had contraindications for a Fontan operation and were offered heart transplantation instead were not included in the calculations [11–13]. If the outcomes of all pre-Fontan operations had been included in the calculations of Fontan outcomes (i.e. if data were collected on an intention-to-treat basis), these would likely be worse. Conversely, data on the outcomes of biventricular repair are collected on an intention-to-treat basis, starting from the first procedure in the neonatal period. Therefore, the two groups are not comparable. In addition, the comparison should not be done with Fontan outcomes in general but specifically with Fontan outcomes in patients with a relatively large rudimentary ventricle, since such are the patients in question here and recent data shows that such patients do worse than usual with a Fontan circulation [14, 15]. However, the biventricular strategy does result in an excellent outcome (practically a cure) in a minority of patients (15% in this series); the challenge is in predicting which specific patients would respond to this strategy so well so that this may be offered selectively to them.

Such predictive factors have been searched for in several studies, and various factors have been suggested based on ventricular volumes, stroke volume, end-diastolic pressure, valvar sizes and various ventricular shape criteria. However, none of these have been found fully reliable [2–8]. The only reliable LV factor has been the presence or absence of endocardial fibroelastosis, but this by itself is insufficient for decision making [4, 5]. However, an interesting recent finding is that right ventricular parameters may be more significant predictors of outcome, with larger right ventricles being associated with worse outcomes [4]. This finding may possibly be explained by the fact that both ventricles support the systemic circulation prior to biventricular repair whereas, after repair, the already-limited borderline LV will have to do so alone, which represents a relative loss of pumping power; the larger the right ventricle is, the greater the loss of pumping power. But this factor has not yet been adequately studied to see whether it really can be a useful predictor of outcome. Therefore, the search continues, and the idea has emerged that perhaps the essential predictors of success are not related to ventricular size but factors within myocardial cells which remain to be explored [4]. This idea is somewhat supported by the fact that the outcome of biventricular repair in hearts with a different malformation, namely double inlet ventricles (ventricular septation procedures), appears to be better, with lower mortality rates and better ventricular function amongst survivors. However, such cases are prone to other complications, especially a significant rate of atrioventricular conduction block requiring implantation of a permanent pacemaker [24–26].

These struggles and disappointments with biventricular repair of hearts with a borderline LV have prompted the use of an alternative strategy for biventricular repair, namely Ventricular Switch [27–29]. This strategy proposes writing off the LV as the systemic ventricle altogether and altering its connections such that it supports the pulmonary circulation instead, which is much less demanding, and setting up the right ventricle, which is normal and has usually maintained its muscle mass in such conditions, to support the systemic circulation. Of note, however, if doubt exists regarding the ability of such a left ventricle to support even the pulmonary circulation, Ventricular Switch may be combined with a superior cavopulmonary anastomosis, thus establishing a “one-and-a-half ventricle repair”, which would make the work of the left ventricle easier in supporting the pulmonary circulation. Ventricular Switch is not one specific operation but a combination of surgical reconstructions determined by the measures required to achieve this objective in each case, which may or may not be done in one stage. Therefore, the operations may vary considerably between patients. This strategy was developed primarily for biventricular repair of complex malformations in which the LV could not be connected easily to the aorta because of various anatomic constraints, but it has also been used in the management of hearts with a borderline LV. Either way, this strategy is usually technically complex and therefore potentially high-risk with its own list of challenges and possible complications [30]. In addition, it has been well shown that the right ventricle is not an ideal systemic pump in the long term, but the outcome with the Ventricular Switch is theoretically expected to be better than with a Fontan circulation [27–29]. This theoretical expectation is supported by analogy with patients with congenitally corrected transposition of the great arteries, who also have a systemic right ventricle but live better and longer than those with a Fontan circulation. In addition, this avoids the usual complications of a Fontan circulation, which can increase the risk of heart transplantation if and when this becomes necessary. However, this optimistic expectation does not take into account the complexity and potential complications of Ventricular Switch, which may compromise outcome. This is illustrated in a recent report of 17 cases of Ventricular Switch showing a high mortality: operative mortality of 18% and 1-year and 5-year mortalities of 21% and 28% respectively [31].

Mortality apart, avoidance of the morbidities of the Fontan circulation is often mentioned as a major advantage of adopting a biventricular strategy. These morbidities include hepatic dysfunction, renal dysfunction, lymphatic dysfunction, plastic bronchitis, protein-losing enteropathy and limited exercise capacity due to inability to increase cardiac output. However, the biventricular repair strategy also often causes significant complications and morbidities as outlined above, although these are different from those of the Fontan circulation, which makes comparisons difficult such as in deciding which complication is worse than which and in comparing their incidences.

The methodology of this work requires some clarification, specifically regarding which residual lesions we focused on. We focused on LV dysfunction and small mitral and aortic valves as the crucial residual lesions. The other lesions or complications are mentioned, especially since they required specific procedures as part of their treatment, but we did not count them as determinants of whether the biventricular repair strategy was considered successful or not. This is because LV dysfunction and small mitral and aortic valves are problems that do not have easy or safe solutions and are likely to undermine long-term outcome whereas all the other above-mentioned lesions and complications may be treated successfully with established techniques. Also, LV dysfunction and small valves are problems only because the biventricular approach was used and would be practically irrelevant in the univentricular path where the right ventricle supplies the circulation. Conversely, the other above-mentioned residual lesions or complications could arise just as easily in univentricular palliation such as the need for balloon dilatation of pulmonary arteries or re-coarctation, i.e. these are not problems that are caused specifically by opting for biventricular repair. The only exception to this is atrioventricular conduction block requiring a permanent pacemaker which is far more likely to occur with certain biventricular repairs than with univentricular palliation, but this does have an easy solution.

This study has important limitations that must be recognized. First, it addresses an uncommon clinical situation which results in the cohort being small and spread over a long period. Second, it is retrospective which severely limits what data are available including and especially the exact criteria used for both the diagnosis of a borderline LV and the decision to opt for a biventricular strategy which may have varied between patients. Third, for all the above reasons, this study does not provide enough information for a search and analysis of potential predictors of outcome, nor to analyze any potential era effect. Nevertheless, just by looking at the Supplemental File––Table 1, there seems to be no era effect; the results, both good and bad, seem to be distributed evenly over the years.

Despite these limitations, however, two important observations may be made:

1. Overall, the outcomes are suboptimal, but.
2. The outcome is excellent in a minority of patients.

This clearly demonstrates that a strategy of biventricular repair in patients with borderline LVs should not be forced upon all patients and that we must continue to look for ways of identifying those who would benefit most from it and offer it to them selectively.

Lastly, it must be emphasized that this study cannot determine whether univentricular palliation would have produced better outcomes in the same patients. Our data only show that a biventricular strategy in a borderline LV population carries a high risk and that improved selection criteria are needed rather than implying superiority of one pathway over another.

## Supplementary Information

Below is the link to the electronic supplementary material.

Supplementary Material 1.

## Data Availability

All data supporting the findings of this study are available within the paper and its supplementary file.
