# Supplementary material for: Outcome of biventricular repair of congenital cardiac malformations with a borderline left ventricle
Source: J Cardiothorac Surg. 2026 Jul 17;21:772. doi: 10.1186/s13019-026-04531-w (PMC13659989; doi:10.1186/s13019-026-04531-w)
Supplement: Supplementary file 1 [file 13019_2026_4531_MOESM1_ESM.docx]

**Table 1:	Patients’ diagnoses, treatments and outcome**

ASD: Atrial septal defect

ECMO: Extracorporeal membrane oxygenation

LA: Left atrium

LAP: Left atrial pressure

LV: Left ventricle

LVEDP: Left ventricular end-diastolic pressure

LVOTO: Left ventricular outflow tract obstruction

RV: Right ventricle

VSD: Ventricular septal defect

Year: Year of birth and first intervention (the first intervention in all patients took place in the neonatal period)

**Year	Diagnosis	Operations	Outcome**

**–––––––––––––––––––––––––––––––––––––––––––––––––––––––––––––––––––––––––––––––––––––––––––––––––––––––––––––––––––––––––––––––––––––––––––**

2006	Bicuspid aortic valve, Z-score: -3	Aortic valve commissurotomy, maintaining a small ASD	Now age 20 years

Mitral valve Z-score: -4	Poor progress	Fontan-associated

LV volume 17 ml/m^2^	Converted to Norwood 3 weeks later	hepatopathy (FALD)

Then Glenn

Multiple catheterizations for embolization of collaterals

Then Fontan. Then, 2 years later: pacemaker implantation

–––––––––––––––––––––––––––––––––––––––––––––––––––––––––––––––––––––––––––––––––––––––––––––––––––––––––––––––––––––––––––––––––––––––––––

2006	Coarctation + hypoplastic aortic arch (subjective)	Coarctectomy + arch augmentation	Now 19 years’ old

LV, aortic valve and mitral valve all subjectively borderline but	Twice balloon dilatation of re-coarctation	NYHA class I

no numerical data available	Mild mitral stenosis

and re-coarctation

**–––––––––––––––––––––––––––––––––––––––––––––––––––––––––––––––––––––––––––––––––––––––––––––––––––––––––––––––––––––––––––––––––––––––––––**

2006	Coarctation + hypoplastic aortic arch (subjective)	Coarctectomy + arch augmentation keeping a small ASD	Now age 20 years

Aortic valve Z-score: -2	Balloon dilatation of re-coarctation	NYHA class I

Mitral valve Z-score: -3	Mild mitral stenosis and

LV volume: 15 ml/m^2^	regurgitation

LV dysfunction

2007	Coarctation + hypoplastic aortic arch (subjective)	Coarctectomy + arch augmentation keeping a small ASD	Now age 19 years, NYHA I

LV volume 11 ml/m^2^	Balloon dilatation of re-coarctation	Mild mitral stenosis with

Mitral valve subjectively small	Z-score: -1.7

**–––––––––––––––––––––––––––––––––––––––––––––––––––––––––––––––––––––––––––––––––––––––––––––––––––––––––––––––––––––––––––––––––––––––––––**

2011	Coarctation + hypoplastic aortic arch	Hybrid palliation (bilateral banding + ductal stent)	Now age 15 years, NYHA II

LV volume 13 ml/m^2^	Later balloon aortic valvuloplasty	Stenotic pulmonary arteries

Mitral and aortic valve Z-scores: -5	Later biventricular conversion, postoperative ECMO	Mild mitral stenosis

Later multiple catheter angioplasty of pulmonary arteries	Free pulmonary regurgitation

Later re-operation to augment the aortic arch	Still has borderline LV

Later Ross-Konno together with re-augmentation of the arch	LV diastolic dysfunction

**–––––––––––––––––––––––––––––––––––––––––––––––––––––––––––––––––––––––––––––––––––––––––––––––––––––––––––––––––––––––––––––––––––––––––––**

2012	Coarctation + hypoplastic aortic arch (subjective)	Coarctectomy + arch repair, maintaining a small ASD	Now 14 years old, NYHA I

Bicuspid aortic valve, subjectively small	Initially severe LV dysfunction, largely recovered	Acceptable LV function

Borderline LV: volume 14 ml/m^2^ but apex-forming	Later, balloon dilatation of re-coarctation

**–––––––––––––––––––––––––––––––––––––––––––––––––––––––––––––––––––––––––––––––––––––––––––––––––––––––––––––––––––––––––––––––––––––––––––**

2013	Coarctation + hypoplastic aortic arch (subjective)	Coarctectomy	Now 12 years’ old, NYHA I

Aortic valve Z-score: -3.7	Slight aortic and mitral

Mitral valve Z-score: -3.1	stenosis but LV grew well

Non-apex forming LV, with a volume of 10 ml/m^2^

**–––––––––––––––––––––––––––––––––––––––––––––––––––––––––––––––––––––––––––––––––––––––––––––––––––––––––––––––––––––––––––––––––––––––––––**

2014	Coarctation + hypoplastic aortic arch (subjective)	Coarctectomy + arch augmentation	Now 9 years old, NYHA I

Borderline LV but no numerical data provided	Good LV size and function

**–––––––––––––––––––––––––––––––––––––––––––––––––––––––––––––––––––––––––––––––––––––––––––––––––––––––––––––––––––––––––––––––––––––––––––**

2014	Coarctation + hypoplastic aortic arch	Coarctectomy, augmentation of aorta and arch, VSD	Died as a neonate

Posterior deviation of outlet septum, small VSD	closure. Not tolerated: converted to Norwood, which

Aortic valve and ascending aorta Z-score -2.5	was not tolerated either: required ECMO but did not

Non-apex-forming LV. LV/RV length ratio 0.85	recover

**–––––––––––––––––––––––––––––––––––––––––––––––––––––––––––––––––––––––––––––––––––––––––––––––––––––––––––––––––––––––––––––––––––––––––––**

2015	Trisomy 21. Coarctation + hypoplastic aortic arch (subjective)	Coarctectomy + arch augmentation.	1 year later: LV not grown

Non-apex forming LV. LV/RV: 0.8.	1 year later: Percutaneous gastrostomy	Pulmonary hypertension

LV diastolic diameter Z-score: -2.4	Died at age 18 months

**–––––––––––––––––––––––––––––––––––––––––––––––––––––––––––––––––––––––––––––––––––––––––––––––––––––––––––––––––––––––––––––––––––––––––––**

2015	Coarctation + hypoplastic aortic arch (Z-score -2.8)	Coarctectomy + arch augmentation.	Currently at age 8 years:

LV diameter Z-score: diastolic -2.8, systolic -3.2	11 months later: Resection of supra-mitral membrane	NYHA class I

Aortic valve Z-score: -2.5	At age 3 years: re-resection of supra-mitral membrane and	LV grown: Z-score: +0.27

Normal mitral valve	aortic valve repair	Mean mitral gradient: 8-10

mmHg. Dilated LA

**–––––––––––––––––––––––––––––––––––––––––––––––––––––––––––––––––––––––––––––––––––––––––––––––––––––––––––––––––––––––––––––––––––––––––––**

2016	Coarctation + hypoplastic aortic arch (subjective)	Coarctectomy + arch augmentation	Now age 9 years

Accessory subaortic mitral valve tissues narrowing the outflow	9 months later: resection of subaortic stenosis	Neurodevelopmental delay

Parachute mitral valve Z-score: -2.3	5 years later: repeat resection of subaortic membrane +	Cerebral palsy

Apex-forming LV but with borderline volume (18 ml/m^2^)	mitral commissurotomy	LV hypertrophy + diastolic

LV diastolic diameter Z-score: -2	dysfunction

Aortic valve subjectively small	LVOTO: gradient 22 mmHg

Severe LA dilatation

Frequent pleural and pericardial effusions and ascites

On diuretics and beta blockers

NYHA class II

**–––––––––––––––––––––––––––––––––––––––––––––––––––––––––––––––––––––––––––––––––––––––––––––––––––––––––––––––––––––––––––––––––––––––––––**

2016	Coarctation + hypoplastic aortic arch (subjective)	Coarctectomy + arch augmentation + ASD closure	Mild mitral stenosis with

LV does not reach the apex, with LV/RV: 0.9	mean gradient of 3 mmHg

Aortic valve Z-score: -3	LVOTO: mean gradient of

Mitral valve Z-score: -1.5	35 mmHg

LA enlargement

Died suddenly at 9 months

**–––––––––––––––––––––––––––––––––––––––––––––––––––––––––––––––––––––––––––––––––––––––––––––––––––––––––––––––––––––––––––––––––––––––––––**

2016	Coarctation + hypoplastic aortic arch (subjective)	Coarctectomy + aortic arch augmentation.	Severe diastolic dysfunction

Parachute mitral valve Z-score: -2.2	Mitral valve repair (commissurotomy, muscle splitting)	Remained on ventilator

Aortic valve with Z-score: -2	Later mitral prosthesis implanted	Died at age 6 months

Apex-forming LV but subjectively small

**–––––––––––––––––––––––––––––––––––––––––––––––––––––––––––––––––––––––––––––––––––––––––––––––––––––––––––––––––––––––––––––––––––––––––––**

2018	Coarctation + hypoplastic aortic arch (Z-score -3.7)	Coarctectomy + arch augmentation + leaving a small ASD	Now 7 years later:

Parachute mitral valve, Z-score: -2.5	Unstable. Put on ECMO on 3rd postop day. 2 days later:	NYHA class I

Apex-forming LV but diameter Z-score: -1.8	Converted to Norwood with a restrictive ASD.	Normal growth and

Multiple complications, but survived	cognitive function

11 months later, the LV and both mitral and aortic valves	LV diameter Z-score: +2.5

had grown well such that	No pulmonary hypertension

biventricular conversion was done

**–––––––––––––––––––––––––––––––––––––––––––––––––––––––––––––––––––––––––––––––––––––––––––––––––––––––––––––––––––––––––––––––––––––––––––**

2018	Coarctation + hypoplastic aortic arch (subjective)	Coarctectomy via thoracotomy	Now age 7 years

Small LV (no numerical data) but apex-forming	Then catheterization for dilatation	NYHA class II

Aortic valve Z-score: -3.5	of the aortic valve	LV has grown

Mitral valve Z-score: -3 with restricted leaflet motion	Mean mitral valve gradient of

5 mmHg and aortic 22 mmHg

Has developed LV

endocardial fibroelastosis

**–––––––––––––––––––––––––––––––––––––––––––––––––––––––––––––––––––––––––––––––––––––––––––––––––––––––––––––––––––––––––––––––––––––––––––**

2018	Coarctation + hypoplastic aortic arch (subjective)	Hybrid palliation (bilateral banding + ductal stent)	Now age 7 years, NYHA II

Borderline LV but apex-forming, with volume of 26 ml/m^2^	4 months later: LV size was considered improved	Mitral stenosis: mean gradient

Aortic valve Z-score: -5.2	Biventricular conversion with arch reconstruction	of 5-6 mmHg, and mild MR

Small VSD	+ VSD closure + mitral commissurotomy and	Small subaortic membrane

Parachute mitral valve, Z-score: -3	papillary muscle splitting

**–––––––––––––––––––––––––––––––––––––––––––––––––––––––––––––––––––––––––––––––––––––––––––––––––––––––––––––––––––––––––––––––––––––––––––**

2020	Coarctation + hypoplastic aortic arch (subjective)	Norwood with a restrictive ASD	Alive and well 6 years later

Parachute mitral valve Z-score: -3	Biventricular conversion 14 months later	NYHA class I

Bicuspid aortic valve with Z-score: -1.5	(LV had grown to normal size, with Z-score: +1.1)

Non-apex-forming LV. LV/RV length ratio 0.85

**–––––––––––––––––––––––––––––––––––––––––––––––––––––––––––––––––––––––––––––––––––––––––––––––––––––––––––––––––––––––––––––––––––––––––––**

2020	Coarctation + hypoplastic aortic arch (subjective)	Coarctectomy	Died as a neonate

Non-apex forming LV, with volume of 18 ml/m^2^	Then Mitral commissurotomy + enlargement of the

Mitral valve subjectively small	Sinotubular junction. Poor progress

Supra-aortic stenosis (no gradient given)	Converted to Norwood, with postoperative ECMO

Did not recover

**–––––––––––––––––––––––––––––––––––––––––––––––––––––––––––––––––––––––––––––––––––––––––––––––––––––––––––––––––––––––––––––––––––––––––––**

2021	Coarctation + hypoplastic aortic arch (subjective)	Coarctectomy and aortic arch augmentation	At 3.5 years: valves grown

Mitral valve Z-score: -3.	Then balloon dilatation of the zone of repair	Mitral Z-score: - 1.4 with

Bicuspid aortic valve with Z-score: -2.3	mean gradient: 5 mmHg

LV diastolic diameter Z-score: -1.4	Aortic Z-score: +1.2 with

mean gradient: 20 mmHg

NYHA class II

**––––––––––––––––––––––––––––––––––––––––––––––––––––––––––––––––––––––––––––––––––––––––––––––––––––––––––––––––––––––––––––––––––––––––**

2021	Coarctation + hypoplastic aortic arch (subjective)	Norwood, then Glenn, then staged biventricular	Died at age 3 years

Monocuspid aortic valve Z-score between -2.7 and -3.7	conversion, which was not tolerated. Converted

Mitral valve Z-score: -1.7	back to univentricular configuration + mitral valve

LV not reaching apex: LV/RV: 0.90	prosthesis. Required ECMO. Did not recover

**–––––––––––––––––––––––––––––––––––––––––––––––––––––––––––––––––––––––––––––––––––––––––––––––––––––––––––––––––––––––––––––––––––––––––––**

2021	Coarctation + hypoplastic aortic arch (Z-score -3.7)	Hybrid palliation	(bilateral banding + ductal stent)	Now at age 5 years: NYHA I

Apex-forming LV but diameter Z-score : -1.5	At 5 months: Left heart had grown well:	Good growth and cognitive

Mitral valve Z-score: -2.4	Biventricular conversion	development. But severe LV

Aortic valve Z-score: -4.5	2 years later: severe aortic and subaortic stenosis	dilatation with ejection

Ross-Konno done: heart block: pacemaker implanted	fraction of 55% and

mean mitral gradient 6 mmHg

Pulmonary hypertension

**–––––––––––––––––––––––––––––––––––––––––––––––––––––––––––––––––––––––––––––––––––––––––––––––––––––––––––––––––––––––––––––––––––––––––––**

2021	Coarctation + hypoplastic aortic arch (subjective)	Coarctectomy + arch augmentation	Now age 4 years: NYHA I

Borderline LV with volume 18-20 ml/m2	Later: balloon dilatation of re-coarctation	LV grown but with

Aortic valve Z-score: -4.1	diastolic dysfunction

Mitral Z-score: -2.8	LVEDP and LAP 18 mmHg

**–––––––––––––––––––––––––––––––––––––––––––––––––––––––––––––––––––––––––––––––––––––––––––––––––––––––––––––––––––––––––––––––––––––––––––**

2021	Coarctation + hypoplastic aortic arch (subjective)	Coarctectomy + arch augmentation + leaving a small ASD	Now age 4 years, NYHA II

Aortic valve Z-score: -2.4	3 months later: resection of subaortic membrane + aortic	Mean mitral valvar gradient 5

Slightly small mitral valve but non-stenotic	valve commissurotomy	mmHg and aortic 16 mmHg

LV diameter Z-score: -3	LV grown: Z-score: -1.1

with hypertrophy + diastolic

dysfunction with LVEDP of

16 mmHg

**–––––––––––––––––––––––––––––––––––––––––––––––––––––––––––––––––––––––––––––––––––––––––––––––––––––––––––––––––––––––––––––––––––––––––––**

2022	Coarctation + small aortic valve, ascending aorta and arch	Coarctectomy + ascending aorta and arch augmentation	Now age 4 years: NYHA I

LV diameter Z-score: -2.4	4 months later: Percutaneous dilatation of residual stenosis	diastolic dysfunction

LV not reaching apex (LV/RV: 0.9)	which caused a large pseudoaneurysm without correcting	Ascending aorta is still small

the stenosis	LV diameter Z-score: -1.6

Reoperated urgently to repair the pseudoaneurysm and	Mean gradient across the

enlarge the more proximal part of the arch and distal	LVOT and ascending aorta

ascending aorta.	of 40 mmHg

**–––––––––––––––––––––––––––––––––––––––––––––––––––––––––––––––––––––––––––––––––––––––––––––––––––––––––––––––––––––––––––––––––––––––––––**

2022	Coarctation + hypoplastic aortic arch (subjective)	Hybrid palliation (Bilateral banding + ductal stent)	Died at age 3 years

LV does not reach the apex: LV/RV: 0.9	Anatomic repair at age 10 months, required ECMO

Aortic valve Z-score: -3.4	Multiple complications on ECMO but recovered.

Normal mitral valve with Z-score: +0.3	While on ECMO: Stenting of the right pulmonary artery

Later, more catheter interventions

Ross-Konno at age 3 years

Good immediate result of Ross-Konno but 10 days later

became unstable and rapidly deteriorated requiring ECMO.

Suffered a severe cerebral hemorrhage.

**–––––––––––––––––––––––––––––––––––––––––––––––––––––––––––––––––––––––––––––––––––––––––––––––––––––––––––––––––––––––––––––––––––––––––––**

2024	Coarctation + hypoplastic aortic arch (subjective)	Coarctectomy + arch augmentation	Slight aortic stenosis

LV diameter Z-score: -1.2	Mean gradient 16 mmHg

Aortic valve Z-score: -4.4	Mild mitral stenosis

Mitral valve Z-score: -3.6	NYHA class I

**–––––––––––––––––––––––––––––––––––––––––––––––––––––––––––––––––––––––––––––––––––––––––––––––––––––––––––––––––––––––––––––––––––––––––––**
